# Permittivity of ex vivo healthy and diseased murine skeletal muscle from 10 kHz to 1 MHz

**DOI:** 10.1038/s41597-019-0045-2

**Published:** 2019-04-18

**Authors:** J. A. Nagy, C. J. DiDonato, S. B. Rutkove, B. Sanchez

**Affiliations:** 1 Department of Neurology, Beth Israel Deaconess Medical Center, Harvard Medical School, Boston, MA 02115 USA; 2 Human Molecular Genetics Program, Stanley Manne Research Institute, Ann & Robert H. Lurie Children’s Hospital, Chicago, IL 60611 USA; 3 Department of Pediatrics, Feinberg School of Medicine, Northwestern University, Chicago, IL 60611 USA

**Keywords:** Neuromuscular disease, Computational biophysics, Mouse

## Abstract

A better understanding of the permittivity property of skeletal muscle is essential for the development of new diagnostic tools and approaches for neuromuscular evaluation. However, there remain important knowledge gaps in our understanding of this property in healthy and diseased skeletal muscle, which hinder its translation into clinical application. Here, we report the permittivity of gastrocnemius muscle in healthy wild type mice and murine models of spinal muscular atrophy, muscular dystrophy, diabetes, amyotrophic lateral sclerosis and in a model of myofiber hypertrophy. Data were measured ex vivo from 10 kHz to 1 MHz using the four-electrode impedance technique. Additional quantitative histology information were obtained. Ultimately, the normative data reported will offer the scientific community the opportunity to develop more accurate models for the validation and prediction of experimental observations in both pre-clinical and clinical neuromuscular disease research.

## Abstract (summary)

**Table:** 

Design Type(s)	physiological data analysis objective • strain comparison design • ex vivo design
Measurement Type(s)	permittivity property
Technology Type(s)	impedance analyzer
Factor Type(s)	temporal_instant • frequency • Mouse Model • experimental condition
Sample Characteristic(s)	Mus musculus • skeletal muscle tissue

Machine-accessible metadata file describing the reported data (ISA-Tab format)

## Background & Summary

Electromagnetism constitutes a basic physical principle widely used in the field of biomedical engineering, designed to monitor and treat a broad spectrum of conditions including Parkinson’s disease^1^ and brain tumors^2,3^. Understanding how different biological tissues and fluids interact with electromagnetic fields is essential for improving the accuracy of existing analytical techniques as well as developing new diagnostic tools and therapies.

In electromagnetism, permittivity is one fundamental material parameter affecting the propagation of electromagnetic fields. When exposed to an electromagnetic field, the dipole moment of the material’s molecules opposes the external electric field and so the net electric field is reduced within the material. In other words, the permittivity is a measure of the ability to store an electric charge in the polarization of the material.

Basic and applied scientific endeavors have reported the permittivity property for well over 100 years in a collective effort to understand the propagation of electromagnetic fields in the human body^4–13^. The birth of biomedical engineering and the field of electrophysiology arose from these pioneers’ early multidisciplinary studies applying new theory, tools and methods. For example, voltage clamp and telegrapher’s equations made it possible to understand how ionic currents give rise to the action potential^14^. Another prominent contribution that emerged from studying tissues’ permittivity includes the development of the bidomain model to study cardiac muscle, which laid the foundation for the development of implantable cardiac pacemakers.

Previous studies have reported permittivity values of specific tissue as a function of the applied frequency. However, a recent meta-analysis revealed that major gaps in the knowledge of the frequency-dependence of the conductivity and relative permittivity in many tissues still exist^15^, especially for those tissues such as skeletal muscle, in which these properties are directionally dependent^16–20^. For example, prior authors acknowledged the technical limitations of their reported paravertebral muscle permittivity values nominally measured along and across the muscle fiber directions^21^.

In addition, some previous studies of the permittivity property of biological specimens did not specify the state of the tissue examined, even though it is known that the permittivity values change postmortem^22^ and with temperature^23^, nor did they specify the extent of disease, if any, present, and some did not include healthy control tissue for comparison. Other noteworthy factors that have not been exhaustively evaluated include the variation of tissues’ permittivity with age, gender, and disease progression. This missing information highlights critical gaps in our understanding of the factors that affect the permittivity property of biological tissues required to aid in identification of clinically abnormal results in pathological tissue.

The present study is motivated by the need to obtain permittivity values from both healthy and diseased skeletal muscle to aid in the diagnosis and monitoring of patients with neuromuscular disorders (NMDs). Analysis of muscle histology performed over the course of disease progression has shown that the composition and structure of skeletal muscle tissue changes as a function of the specific NMD. For example, in amyotrophic lateral sclerosis (ALS) muscle fibers tend to atrophy over time^24^, whereas in Duchenne muscular dystrophy, the lack of dystrophin protein^25^, causes a loss of muscle fibers and their progressive substitution by fat and fibrous tissue^26^.

Changes in the permittivity property in diseased muscle establishes the underlying scientific premise of electrical impedance myography (EIM)^27,28^, a relatively new electrodiagnostic technique that is gradually finding its niche for the assessment of NMD progression and the success of therapeutic intervention^29^. EIM is a non-destructive technique that is based on the measurement of the electrical impedance of individual muscles or groups of muscles. The permittivity property of the muscle can be extracted from the EIM values by accounting for the experimental setup. To date, however, EIM studies that have reported permittivity values of diseased muscle are scarce and often the data are incomplete, evaluating permittivity values at only a single frequency^30^. For EIM to achieve its full potential, it is paramount to first establish normative permittivity values in the frequency range where the histological alterations in disease would be expected to have an effect.

Here, using EIM technique, we report the conductivity and relative permittivity values of mouse muscle in healthy and four disease models (i.e., spinal muscular atrophy, muscular dystrophy, diabetes, amyotrophic lateral sclerosis) and in a drug-induced model of myofiber hypertrophy, which represents an all-encompassing effort by our group over the course of the last three years. The objective of this report is to equip the reader with a comprehensive database of the permittivity properties of healthy and diseased skeletal muscle from 10 kHz to 1 MHz, in both longitudinal and transverse directions. We also include additional quantitative histology data and periodic measurements obtained during disease progression. Providing the permittivity of healthy and diseased muscle using these various mouse models will open new venues for the development and improvement of the clinical diagnosis and monitoring of patients with NMD.

## Methods

### Terminology and definitions

The permittivity *ε* determines the dielectric behavior of materials when exposed to an applied electric field and it is defined as (dimensionless)

1 $$\varepsilon ={\varepsilon }_{r}-i\frac{\sigma }{\omega {\varepsilon }_{0}},$$

where *ε*_r_ is the relative permittivity (dimensionless), *σ* is the total conductivity of the material (S m^−1^), *ω* is the (angular) frequency of the field measured (rad s^−1^), *ε*_0_ = 8.85 · 10^−12^ is the permittivity of the vacuum (F m^−1^), and *i* is the imaginary unit (dimensionless).

In skeletal muscle, due to its highly organized cellular and fascicular structure, the permittivity is different along and perpendicular to the direction determined by the myofibers orientation^31^. Here, we calculated the longitudinal and transverse conductivity and relative permittivity from longitudinal and transverse resistance R and reactance X muscle data measured using a dielectric cell with the four-electrode technique,

2 $${\sigma }_{\{L,T\}}=K\frac{{R}_{\{L,T\}}}{{R}_{\{L,T\}}^{2}+{X}_{\{L,T\}}^{2}}\,and\,{\varepsilon }_{r,\{L,T\}}=K\frac{{X}_{\{L,T\}}}{\omega {\varepsilon }_{0}\left({R}_{\{L,T\}}^{2}+{X}_{\{L,T\}}^{2}\right)},$$

where *K* (m^−1^) is a geometrical factor determined from measurements in saline solution.

### Ethical approval and informed consent

All animal procedures were carried out in strict accordance with the recommendations in the Guide for the Care and Use of Laboratory Animals of the NIH and approved by the Institutional Animal Care and Use Committee at Beth Israel Deaconess Medical Center (Protocol #087–2016).

### Animal experimentation

Animal experimentation was the same for all animals. All animal procedures were carried out in strict accordance with the recommendations in the Guide for the Care and Use of Laboratory Animals of the NIH and approved by the Institutional Animal Care and Use Committee at Beth Israel Deaconess Medical Center (Protocol #087–2016). Mice were given ad libitum access to food (Formulab Diet 5008, LabDiet, MO, USA) and water. Animals were allowed to acclimate at least 72 h prior to testing. Prior to measurements, animals were humanely euthanized with CO_2_ and the gastrocnemius muscle harvested from both left and right legs. Muscle impedance measurements were made immediately after with a heating pad under the dielectric cell to maintain a constant temperature 37 °C. Unless otherwise stated, the animals were obtained from Jackson Laboratories (JAX, Bar Harbor, ME, USA).

### Spinal muscular atrophy (SMA) mice

Five spinal muscular atrophy (SMA) Model (“Smn 2B/2B-Neo”) (B6.129-Smn1tm1.1Cdid/tm1Cdid) mice were generated by intercrossing SMN 2B mice with SMN 2B-Neo mice. The official name of the Smn 2B allele is B6.129-Smn1tm1.1Cdid. 2B mice were generated from the progenitor line Smn 2B-Neo (B6.129-Smn1tm1Cdid) through removal of the flox-neo cassette^32^. Germline mice were subsequently crossed to C57BL/6J mice (JAX stock #000664) for at least 3 generations prior to use in these studies. The median survival of 2B/2B-Neo mice is ~13 months for males and 24 months for females. Details of this model will be reported elsewhere. Mice were studied at 40 weeks of age. Five wild type littermates served as controls for EIM analysis.

### Muscular dystrophy (MDX) mice

The D2.B10 (DBA/2-congenic) *Dmd*^*mdx*^ mouse (also referred in the literature as DBA/2J-mdx or D2-mdx mice) was chosen as a model of Duchenne muscular dystrophy model as it recapitulates several of the human characteristics of DMD myopathology including lower hind limb muscle weight, fewer myofibers, increased fibrosis and fat accumulation, and muscle weakness relative to strains with this mutant allele on other genetic backgrounds^33–35^. These genetically altered mice develop the disease without additional intervention and live at least one year. Fifteen male D2.B10-*Dmd*^*mdx*^/J mice hemizygous for *Dmd*^*mdx*^ (6–9 weeks of age, JAX strain #013141) and studied at various ages from 6 to 43 weeks. Fifteen male wild type mice (DBA/2J, JAX strain #000671) served as controls (5 mice per time point).

### Obese/Diabetic mice

The DB/DB mouse (BKS.Cg-Dock7m +/+ Leprdb/J) was chosen as a model of diabetes type II and obesity^36–39^. Mice homozygous for the diabetes spontaneous mutation (Leprdb) become obese at approximately three to four weeks of age. Elevations of plasma insulin begin at 10 to 14 days and elevations of blood sugar at four to eight weeks. Homozygous mutant mice are polyphagic, polydipsic, and polyuric. These mice are well known for their obesity and for developing substantial intramuscular fat deposition by approximately 8 weeks of age. Ten male mice (5 weeks, JAX strain #000642) were studied at 6 and 20 weeks (5 mice per time point). Ten WT type C57BLKS/J (JAX strain #000662) served as controls (5 mice per time point).

### Amyotrophic lateral sclerosis (ALS) mice

Breeding pairs of ALS B6SJL-Tg(SOD1*G93A)1Gur/J mice (JAX strain #002726) were obtained and bred to obtain 37 animals (approximately half female and half male). To study varying fiber size^40^, animals were studied at various ages ranging from 8–18 weeks (approximately 6–7 animals per fortnight, at 8, 10, 12, 14, 16, and 18 weeks).

### Mice with myofiber hypertrophy

Twenty male wild type mice (C57BL/6J, 8 weeks of age, JAX strain #000664) were obtained. Starting at 9 weeks of age, mice were divided randomly into two groups of 10 mice per group. Mice were treated twice weekly with subcutaneous injections of either phosphate-buffered saline (PBS) or the myostatin ligand trap ActRIIB-mFc (Acceleron Pharma, Cambridge, MA, USA) at a dose of 3.3 mg/kg. ActRIIB-mFc (also termed RAP-031)^41^ is a protein comprised of a form of the extracellular domain of ActRIIB fused to a mouse Fc that acts as a ligand trap to inhibit myostatin signaling. Animals were weighed weekly with an analytical balance (AS64, Adventurer SL, Ohaus Corporation, Pine Brook, NJ, USA) to ensure correct dosing throughout the course of the study. All procedures were performed after 5 weeks of treatment with ActRIIB-mFc. Dose-dependent impedance and functional data were published elsewhere^42^.

### *Ex vivo* analysis of excised gastrocnemius muscle

Following euthanasia via CO_2_ inhalation, the gastrocnemius muscle was excised at its proximal extent just below the knee and distally by cutting the gastrocnemius tendon at its insertions, after removing the bicep femoris muscle. Each gastrocnemius muscle was trimmed with a scalpel to 5 mm (width) × 5 mm (length) centered at the belly of the muscle in order to fit into the dielectric measuring cell.

### Dielectric cell

We used the dielectric cell described elsewhere^43^. Briefly, the dielectric cell was made of two flat plate stainless steel electrodes for applying electrical current side to side of the slab of muscle. The voltage electrodes were two monopolar EMG needles in contact the top surface of the muscle (Carefusion #902-DMG50-TP). The geometrical dimensions of the dielectric cell were 5 mm (width) × 5 mm (length), the muscle height measured with a caliper. The distance between the voltage electrodes was 4 mm. The slab of muscle was inserted into the cell first with the muscle fibers oriented parallel (longitudinal) and then perpendicular (transverse) to the current electrodes. The geometric factor *K* was determined from measurements of saline solution with the corresponding height of each tissue sample.

### Four-electrode impedance measurements

Muscle resistance and reactance were collected from 9 to 862 kHz using a commercial impedance analyzer (EIM1103, Myolex, Inc., Boston, MA, USA).

### Statistical analysis

Student’s t-tests, two-tailed, significance p = 0.05 was used to statistically examine the myofiber cross sectional area difference between healthy and diseased muscle tissue as quantified through histological assessment of the muscle.

### Histological analysis

After *ex vivo* EIM measurements were completed, the left gastrocnemius was fixed in 10% formalin, embedded in paraffin and sectioned into 10 *μ*m slices. Sections were stained with collagen VI antibodies (ab6588; Abcam, Cambridge, MA, USA) to identify the cell membrane and with 4′,6-diamidino- 2-phenylindole to stain nuclei. Individual sections were viewed at 20X and photographed with an epifluorescence microscope (Axio Imager M1, Zeiss, Oberkochen, Germany). Myofiber area was identified and analyzed with FIJI (ImageJ, NIH) using the muscle morphometry plug-in (Anthony Sinadinos using Eclipse IDE) to calculate the cross sectional area of the myofibers. On average, ~250 fibers were counted per muscle. Careful attention was paid to measure all cells that appeared to be myofibers, with the goal of measuring representative fields that accurately reflected the overall status of the muscle.

## Data Records

The dataset described is freely available in PhysioNet^44^. The format of the data files is the same for healthy and diseased muscle conditions. The anisotropic permittivity property measured in longitudinal and transverse directions is organized in columns while the frequency dependence is organized in rows. For each time point measured, which also is organized in columns, the permittivity average value and the standard error of the mean are reported.

## Technical Validation

**Fig. 1:**
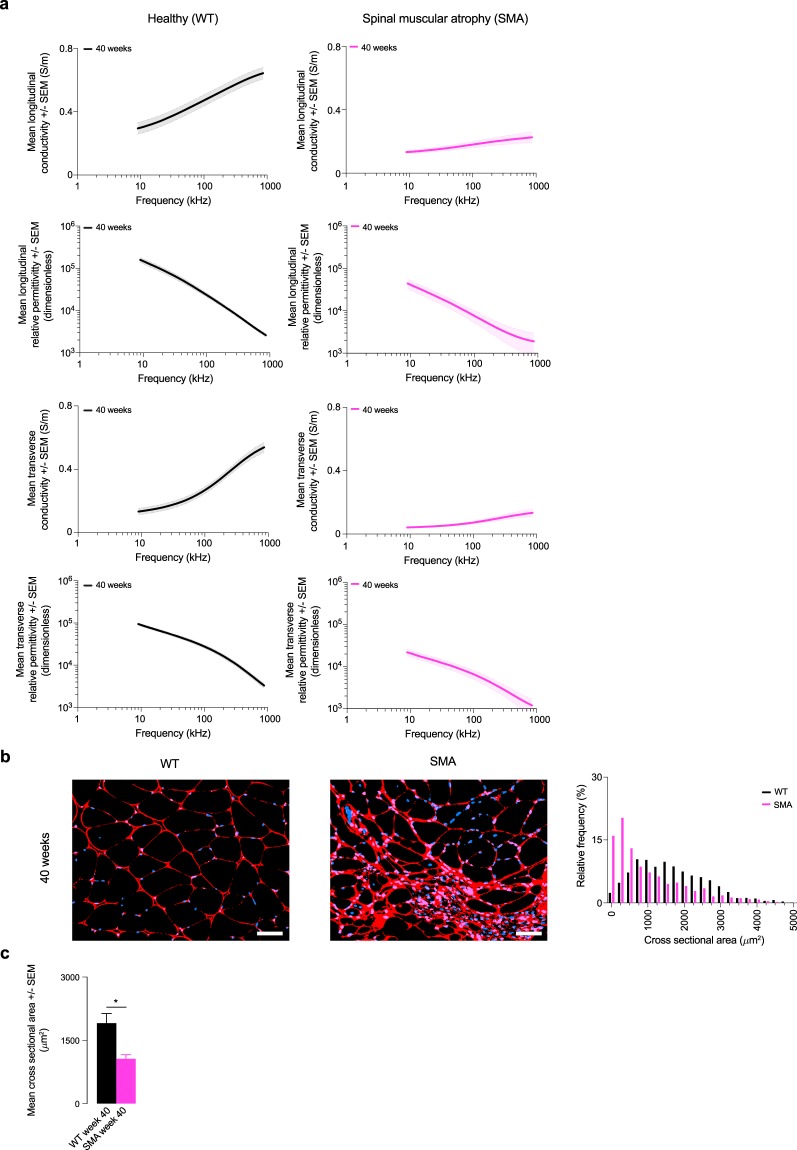
Permittivity in a mice model of spinal muscular atrophy. (**a**) Longitudinal and transverse conductivity and relative permittivity of healthy wild type (WT) and spinal muscular atrophy (SMA) mice. Mean ± standard error of the mean (SEM). (**b**) Representative histology images from the gastrocnemius muscle. Scale bar: 50 *μ*m. Quantification of the myofiber cross sectional area (CSA) in WT and SMA mice. (**c**) Mean CSA. *p < 0.05.

**Fig. 2:**
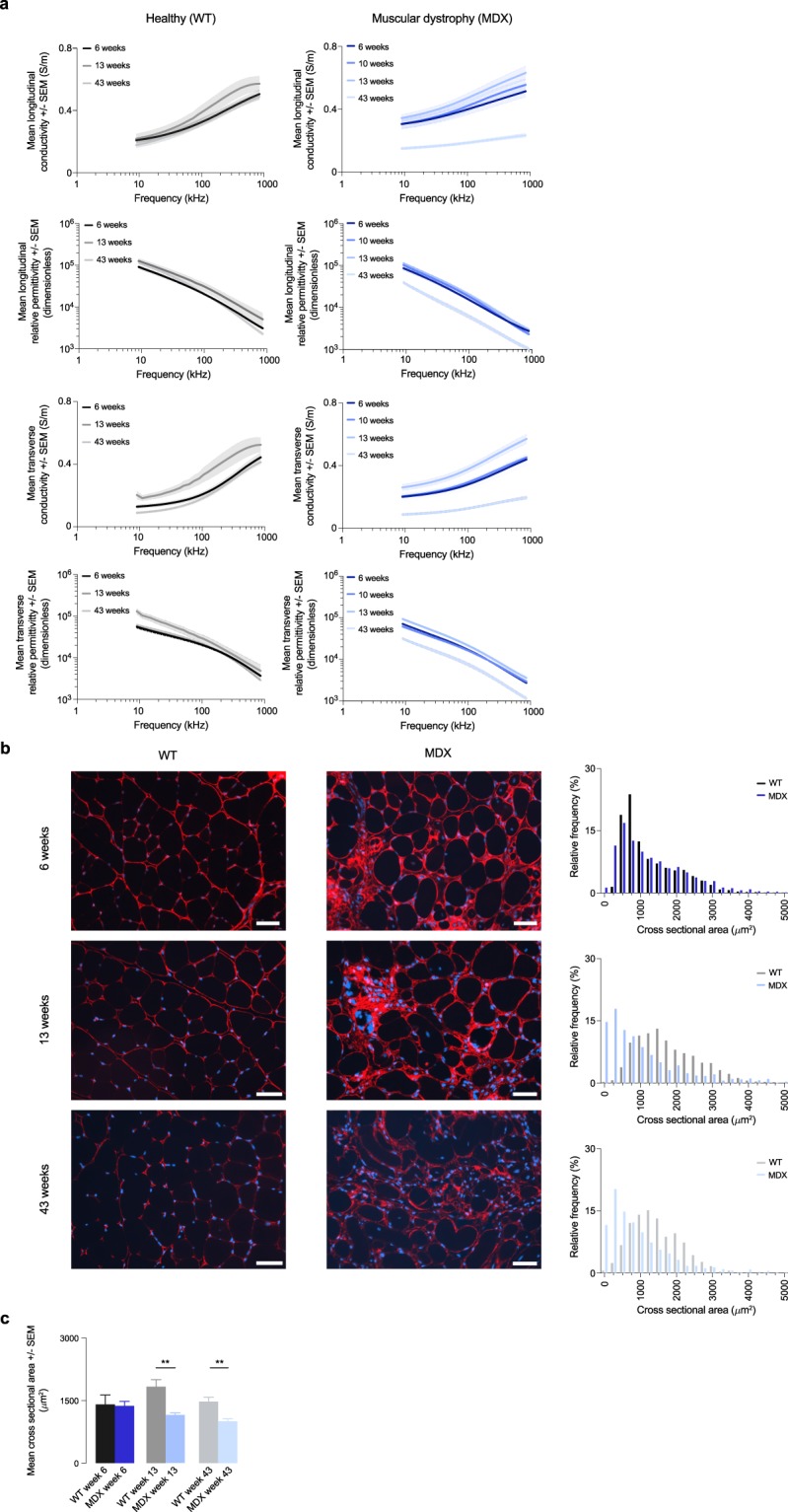
Permittivity in a mice model of muscular dystrophy. (**a**) Longitudinal and transverse conductivity and relative permittivity of healthy wild type (WT) and muscular dystrophy (MDX) mice. Mean ± standard error of the mean (SEM). (**b**) Representative histology images from the gastrocnemius muscle. Scale bar: 50 *μ*m. Quantification of the myofiber cross sectional area (CSA) in WT and MDX mice. (**c**) Mean CSA. **p < 0.01.

**Fig. 3:**
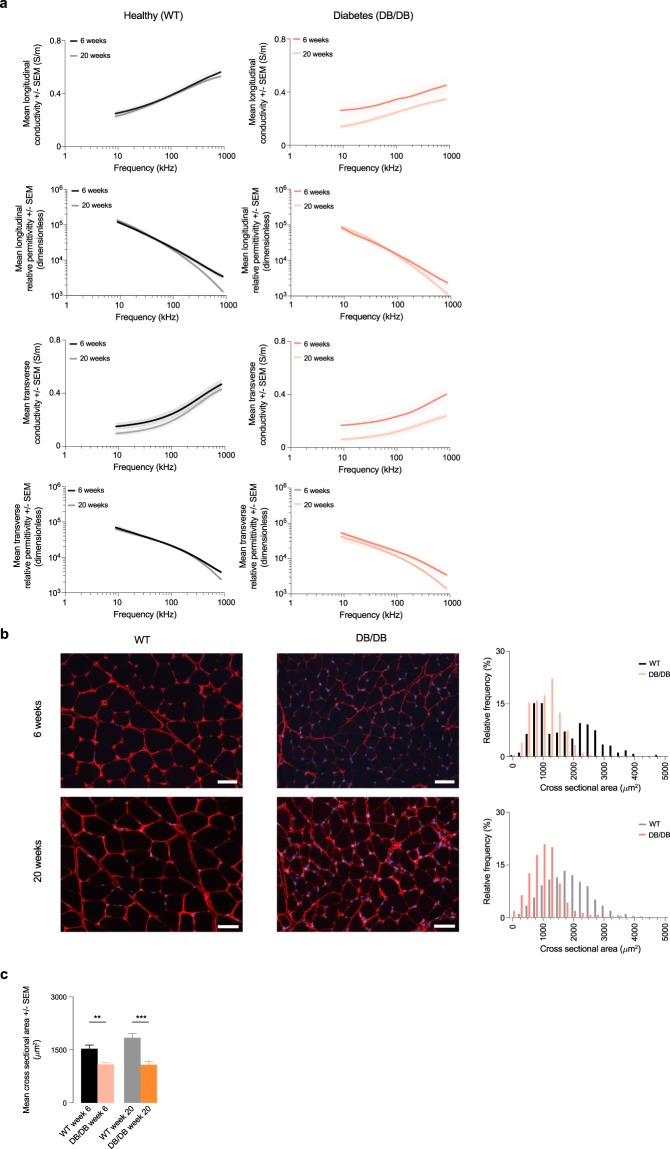
Permittivity in a mice model of diabetes. (**a**) Longitudinal and transverse conductivity and relative permittivity of healthy wild type (WT) and diabetes (DB/DB) mice. Mean ± standard error of the mean (SEM). (**b**) Representative histology images from the gastrocnemius muscle. Scale bar: 50 *μ*m. Quantification of the myofiber cross sectional area (CSA) in WT and DB/DB mice. (**c**) Mean CSA. **p < 0.01; ***p < 0.001.

**Fig. 4:**
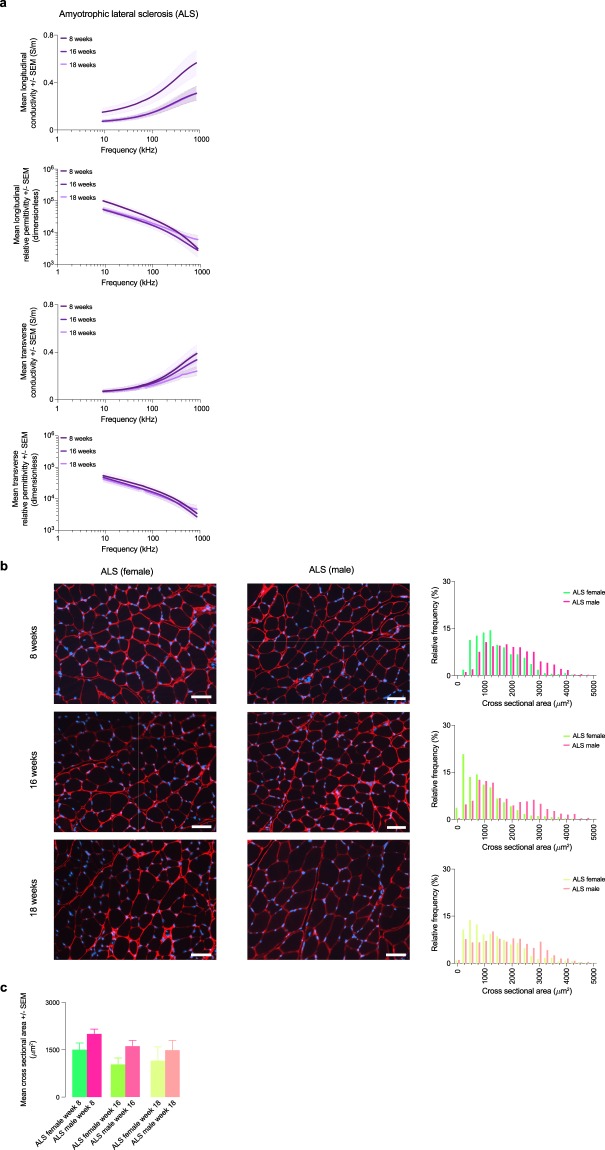
Permittivity in a mice model of amyotrophic lateral sclerosis. (**a**) Longitudinal and transverse conductivity and relative permittivity of male and female amyotrophic lateral sclerosis (ALS) mice. Mean ± standard error of the mean (SEM). (**b**) Representative histology images from the gastrocnemius muscle. Scale bar: 50 *μ*m. Quantification of the myofiber cross sectional area (CSA) in male and female ALS mice. (**c**) Mean CSA.

**Fig. 5:**
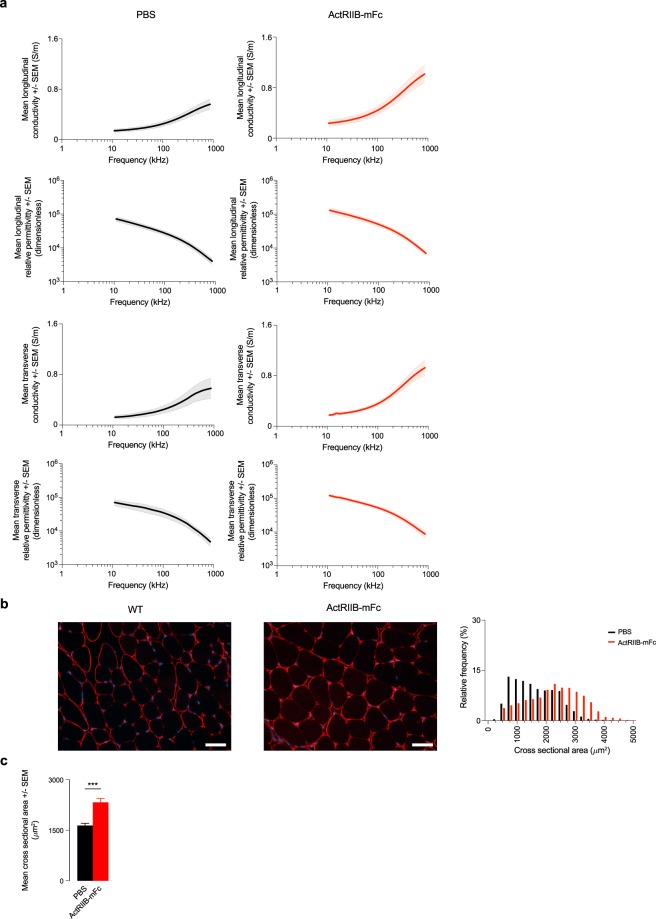
Permittivity in a mice model of myofiber hypertrophy. (**a**) Longitudinal and transverse conductivity and relative permittivity of healthy wild type (WT) mice treated with phosphate-buffered saline (PBS) and myostatin ligand trap ActRIIB-mFc. Mean ± standard error of the mean (SEM). (**b**) Representative histology images from the gastrocnemius muscle. Scale bar: 50 *μ*m. Quantification of the myofiber cross sectional area (CSA) in WT treated with PBS and ActRIIB-mFc. (**c**) Mean CSA. ***p < 0.001.

Our murine data on SMA (Fig. 1), MDX (Fig. 2), DB/DB (Fig. 3), ALS (Fig. 4), and a model of myofiber hypertrophy (Fig. 5) fill the gap of previous studies published in the literature. A single-frequency summary of conductivity values is provided in Table 1 (multi-frequency data are available in PhysioNet). Regarding the relative permittivity (not shown), we found the longitudinal values were greater than in transverse direction in the low frequencies, an observation that had been previously attributed to the polarization of the t-tubule muscle system^20,31,45^.

**Table 1:** Summary of skeletal muscle conductivity values reported at 50 kHz.

	Model (strain)	Time point	Muscle	Direction	Conductivity (S m^−1^)
Li *et al*.^30^	WT (C57Bl/6N)	10 weeks	Mouse gastrocnemius	L	0.5
				T	0.15
	ALS (SOD1*G93A)	18 weeks		L	0.5
				T	0.3
	MDX (10ScSn-DMDmdx/J)	10 weeks		L	0.7
				T	0.4
Epstein *et al*.^31^	WT (n.a.)	n.a.	Canine adductor magnus et brevis	L	0.7
				T	0.1
Sanchez *et al*.^43^	WT (Wistar)	14 weeks	Rat gastrocnemius	L	0.6
				T	0.25
Rush *et al*.^46^	WT (Mongrel)	14–30 kg	Canine longissimus dorsi, spinalis dorsi et cervicis	L	0.35–0.81
				T	0.04–0.1
Ahad *et al*.^47^	WT (Wistar)	140–160 g	Rat immature gastrocnemius	L	0.44 ± 0.04
				T	0.12 ± 0.01
		420–480 g	Rat mature gastrocnemius	L	0.38 ± 0.02
				T	0.15 ± 0.01
	ALS (SOD1*G93A)	8 weeks	Mouse gastrocnemius	L	0.23 ± 0.05
				T	0.11 ± 0.03
		18 weeks		L	0.11 ± 0.02
				T	0.10 ± 0.01
	D2-mdx (DBA/2-congenic)	6 weeks	Mouse gastrocnemius	L	0.36 ± 0.03
				T	0.24 ± 0.03
		10 weeks		L	0.37 ± 0.04
				T	0.25 ± 0.01
		13 weeks		L	0.42 ± 0.03
				T	0.33 ± 0.02
		43 weeks		L	0.17 ± 0.01
				T	0.11 ± 0.01
This work	WT for D2-mdx (DBA/2J)	6 weeks	Mouse gastrocnemius	L	0.28 ± 0.02
				T	0.17 ± 0.01
		13 weeks		L	0.31 ± 0.04
				T	0.26 ± 0.03
		43 weeks		L	0.27 ± 0.02
				T	0.13 ± 0.01
	SMA (Smn 2B/2B-Neo)	40 weeks	Mouse gastrocnemius	L	0.16 ± 0.02
				T	0.06 ± 0.01
	DB/DB (BKS.Cg-Dock7m +/+ Leprdb/J)	6 weeks	Mouse gastrocnemius	L	0.31 ± 0.04
				T	0.20 ± 0.04
		20 weeks		L	0.21 ± 0.01
				T	0.09 ± 0.02
	WT for DB/DB (C57BLKS/J)	6 weeks	Mouse gastrocnemius	L	0.34 ± 0.02
				T	0.20 ± 0.03
		20 weeks		L	0.33 ± 0.01
				T	0.14 ± 0.02
	Mice (C57BL/6J) treated with ActRIIB-m Fc	14 weeks	Mouse gastrocnemius	L	0.34 ± 0.06
				T	0.26 ± 0.02

Abbreviations: L, longitudinal; T, transverse; n.a., not available.

In the absence of *in vivo* human muscle permittivity values, mouse muscle permittivity data reported here allow the user to approximate, through the use of analytic models, EIM values for the conditions studied. It also gives the user the ability to predict the temporal evolution with disease progression using the permittivity from different time points. For example, EIM phase angle *θ* can be computed in the longitudinal and transverse direction as follows

$$\theta \approx -{tan}^{-1}\left(\frac{2\pi f{\varepsilon }_{0}{\varepsilon }_{r}}{\sigma }\right),$$

where *f* is the frequency in Hz. The reader can also use the permittivity reported to perform more accurate numerical simulation studies using finite element models for example.

## ISA-Tab metadata file

Download metadata file

## Data Availability

The algorithm used in this work to convert impedance data to permittivity is described in Eq. 2.
